# Royal Jelly Alleviates Cognitive Deficits and β-Amyloid Accumulation in APP/PS1 Mouse Model Via Activation of the cAMP/PKA/CREB/BDNF Pathway and Inhibition of Neuronal Apoptosis

**DOI:** 10.3389/fnagi.2018.00428

**Published:** 2019-01-04

**Authors:** Mengmeng You, Yongming Pan, Yichen Liu, Yifan Chen, Yuqi Wu, Juanjuan Si, Kai Wang, Fuliang Hu

**Affiliations:** ^1^College of Animal Sciences, Zhejiang University, Hangzhou, China; ^2^Institute of Apicultural Research, Chinese Academy of Agricultural Sciences, Beijing, China

**Keywords:** royal jelly, Alzheimer’s disease, cognitive deficits, amyloid-β, cAMP-response element binding protein, apoptosis

## Abstract

Alzheimer’s disease (AD) is characterized clinically by progressive cognitive decline and pathologically by the accumulation of amyloid-β (Aβ) in the brain. Royal jelly (RJ), a secretion of honeybee hypopharyngeal and mandibular glands, has previously been shown to have anti-aging and neuromodulatory activities. In this study, we discovered that 3 months of RJ treatment substantially ameliorated behavioral deficits of APP/PS1 mice in the Morris Water Maze (MWM) test and step-down passive avoidance test. Our data also showed that RJ significantly diminished amyloid plaque pathology in APP/PS1 mice. Furthermore, RJ alleviated c-Jun N-terminal kinase (JNK) phosphorylation-induced neuronal apoptosis by suppressing oxidative stress. Importantly, hippocampal cyclic adenosine monophosphate (cAMP), p-PKA, p-CREB and BDNF levels were significantly increased in the APP/PS1 mice after RJ treatment, indicating that the cAMP/PKA/CREB/BDNF pathway might be related to the ameliorative effect of RJ on cognitive decline. Collectively, these results provide a scientific basis for using RJ as a functional food for targeting AD pathology.

## Introduction

Alzheimer’s disease (AD) is an age-related neurodegenerative disease characterized by progressive loss of cognitive and memory function (Citron, 2010). AD afflicts more than 45 million individuals worldwide and has an increasing incidence, which has serious adverse impact on the growing elderly population as well as their families (Prince et al., 2015). Currently, mounting evidence has shown that the extracellular deposition of amyloid-β (Aβ) in the brain, which will lead to insoluble senile plaques (SPs), is a critical hallmark of AD (Campion et al., 2016). It has been reported that Aβ induced the progression of other pathological abnormalities, including intraneuronal neurofibrillary tangles (NFTs), which are caused by hyperphosphorylated tau, neuroinflammation, and neuronal loss. Conversely, these pathological events also aggravated the deposition of Aβ and further promoted AD progression (Tanzi and Bertram, 2005; Bettens et al., 2013). Aβ is produced by amyloid precursor protein (APP) through the amyloidogenic pathway. In the first stage of Aβ generation, the proteolytic cleavage of APP by β-secretase (BACE1) produces part-soluble APP peptide-β (sAPPβ) and C-terminal fragment-β (CTFβ), which are further cleaved by γ-secretase to generate hydrophobic Aβ polypeptides (Aβ40 and Aβ42; Dong et al., 2006). In addition, Aβ-degrading enzymes, including insulin-degrading enzyme (IDE) and neprilysin (NEP), are responsible for the clearance of Aβ in the brain. They are capable of cleaving and converting Aβ polypeptide to benign forms (Mukherjee et al., 2000; Shirotani et al., 2001). Hence, it is widely accepted that excessive deposition of Aβ can be explained by the imbalance between formation and degradation (Hardy and Selkoe, 2002).

Currently, treatment strategies targeting the enhancement of brain Aβ clearing activities, such as Aβ immunotherapy, are being extensively studied (Mangialasche et al., 2010). However, the use of therapeutic antibodies may cause a series of side effects, including vasogenic edema (Salloway et al., 2009), microhemorrhage (Nicoll et al., 2003) and neuronal hyperactivity (Busche et al., 2015). In addition, recent documents showed that Aβ immunotherapy could not improve cognitive deficits in AD patients (Doody et al., 2014). Likewise, tau aggregation inhibitors failed to improve the memory and cognitive function during AD progression (Gauthier et al., 2016). In view of the above failures, there is a need for new drugs to inhibit the cognitive deficits of AD and alleviate the associated neuropathology. Several recent studies have shown the positive effects of natural functional foods in cell culture experiments as well as in animal models of AD (Reddy et al., 2018; Youn et al., 2018). Royal jelly (RJ), a bee product produced by the hypopharyngeal and mandibular glands of worker bees, is a traditional functional food (Sabatini et al., 2009). RJ comprises water, protein, carbohydrates, vitamin, lipids, acetylcholine and other bioactive substances that endow RJ with a variety of pharmacological activities, including anti-inflammatory activity (You et al., 2018), anti-oxidative activity (Guo et al., 2009) and anti-aging activity (Honda et al., 2015). Importantly, there are several reports suggesting that RJ has neuromodulatory activity in different animal models. Hattori et al. (2010b) showed that orally administered RJ improved the cognitive impairment of trimethyltin (TMT)-treated mice. Zamani et al. (2012) found a neuroprotective role for RJ in streptozotocin (STZ) induced sporadic AD rat models. Wang et al. (2015) have verified that supplementation with RJ significantly promoted lifespan and stress resistance in *C. elegans*. Their subsequent studies further showed that RJ alleviated Aβ toxicity in *C. elegans*, and revealed the potential function of RJ in AD treatment (Wang et al., 2016). However, it is not clear whether RJ treatment contributes to the alleviation of familial AD. Thus, in this study, we assessed the effects of RJ on AD pathology and cognitive function in APP/PS1 double transgenic mice and further investigated the underlying mechanisms.

## Materials and Methods

### Gas Chromatography (GC) Detection

The content of* trans*-10-hydroxy-2-decenoic acid (10-HDA), 10-hydroxydecanoic acid (10-HDAA) and sebacic acid (SEA) in RJ samples were measured using GC. 0.25 g lyophilized RJ powder was extracted with 20 mL ethanol for 15 min and 5 mL methyl 4-hydroxybenzoate was added as internal standard. Next the ethanol solvent was removed, 1 mL ether was added and filtered through 0.22 μm filter membrane. After evaporation of the ether, 400 μL pyridine and 80 μL N, O-Bis(trimethylsilyl) trifluoroacetamide (BSTFA; Sigma, Kawasaki, Japan) were added, sealed and heated at 60°C for 60 min to derivatize samples. All derivatized extracts were analyzed on Gas Chromatography-Flame Ionization Detector (GC-FID; GC-2010, Shimadzu, Kyoto, Japan) using InertCap-5 column (30 m × 0.25 mm I.D. × 0.25 μm film). One microliter sample was injected with the help of AOC-20i auto injector (Shimadzu, Kyoto, Japan). Nitrogen was used as a carrier gas and flow was kept constant at 1 mL/min. The injector was held at 280°C and worked on split mode (split ratio 10:1). The initial column temperature was 60°C and held for 5 min, then raised to 300°C at 5°C/min and held for 5 min. The detector was performed at 325°C.

### Animals and Treatments

Ten-month-old APP/PS1 transgenic mice with a C57BL/6 background (B6C3-Tg (APPswe, PSEN1dE9) 85Dbo/J) and age-matched C57BL/6 mice were purchased from the Model Animal Research Center of Nanjing University (Nanjing, China). All experiments were approved by the Institutional Animal Care and Use Committee of Zhejiang Chinese Medical University (IACUC Approval No: ZSLL-2017-079) and were performed according to the guidelines from the Laboratory Animal Research Center of Zhejiang Chinese Medical University (Certificate No. SYXK, Zhejiang, 2013-0184, China). The experimental design of behavioral and biochemical analysis is shown in Supplementary Figure S1. We used equal numbers of female and male APP/PS1 mice at the age of 10 months to explore the effects of RJ on AD after 3 months administration. After 1 week of acclimatization, the mice were divided into the following groups: (1) Wt group, wild-type mice were given intragastric administration of saline; (2) Tg group, APP/PS1 transgenic mice were given intragastric administration of saline; (3) TgRJ group, APP/PS1 transgenic mice were given intragastric administration of RJ; and (4) WtRJ group, wild-type mice were given intragastric administration of RJ. Each group had 10 mice. All groups received oral administration of saline or RJ at a dose of 300 mg/kg/d for 3 months. Behavioral analysis was carried out at 54th and 82th day using Morris Water Maze (MWM) test, and at 89th day using step-down passive avoidance test.

### MWM Test

The spatial learning-memory abilities of mice were assessed by MWM test as described previously (Seung et al., 2018). Briefly, a circular water tank (height 40 cm, diameter 100 cm) was filled with water maintained at 22–25°C. The escape platform (height 35 cm, diameter 6 cm) was placed submerged 1–1.5 cm below the surface of water. Soybean milk powder was added to make water opaque before the test. The navigation test was conducted once a day for six consecutive days with one constant hidden platform point in quadrant 2 and three rotational starting points. The limit time was 120 s/trail and the trail will end as soon as mice stayed on the hidden escape platform for 5 s. Escape latency and swim path tracking of each mouse were recorded by a camera mounted above the center of tank. The probe test was carried out 24 h after the last navigation test to evaluate the memory consolidation of mice. During the probe test, the escape platform was removed and mice could swim freely for 120 s. The time spent in the target quadrant and swim path tracking of each mouse were recorded. All data were analyzed using video-tracking software (SMART, version 2.5.15).

### Step-Down Passive Avoidance Test

The step-down passive avoidance test was performed 1 day after the MWM test as described in previous reports with minor modifications (Ballesta et al., 2012). The equipment comprised five plastic black chambers with parallel, stainless-steel grids and a plastic platform was positioned in a corner of each chamber. Before the training session, mice were gently placed on the grid floor and each mouse was given 5 min for adaptation. After that, the electric currents (36 V) were delivered and maintained for 300 s. The mice would jump onto the platform to avoid the electric shock and the number of errors of each mouse (number of times that the mouse stepped down from platform) was recorded. After a 24 h interval, the retention test was carried out and the electric shock was removed, mice were placed on the platform and the step-down latency of each mouse was recorded. The cut-off time in the training session and the retention session was set to 300 s.

### Brain Tissue Preparation

After the behavior tests, the mice were weighed and deeply anesthetized with pentobarbital sodium (45 mg/kg, intraperitoneally). The whole brain tissues were rapidly removed from the skull on ice. They were then cut sagittally into left and right hemispheres. The cortex and hippocampus in the right hemispheres were dissected on ice, snap frozen in liquid nitrogen and stored at −80°C for biochemical analysis. The left hemispheres were fixed in 4% paraformaldehyde for at least 24 h and then embedded with paraffin and cut into 6 μm sections for further staining analysis.

### Histological Examinations

#### Immunohistochemical (IHC) Staining

The methods of IHC staining have been reported previously (Pan et al., 2018). Briefly, sections were blocked with 3% BSA for 30 min, then incubated with primary antibodies overnight at 4°C, including BACE1 (1:100, Santa Cruz Biotechnology, USA), IDE (1:100, Santa Cruz Biotechnology, USA), β-amyloid (B-4, 1:100, Santa Cruz Biotechnology, USA). PBS was used as a negative control instead of the primary antibody. Subsequently, the sections were washed with PBS three times and were incubated with secondary antibody for 1 h at room temperature. Sections were counterstained with hematoxylin and visualized with DAB (ZSJQ, Beijing, China). The captured images were analyzed using Image Pro Plus 6.0 software (Media Cybernetics, Rockville, MD, USA). For quantification, we calculated BACE1, IDE- and β-amyloid-positive areas under 40× magnification (hippocampus) or under 20× magnification (cortex) in three random fields in the brain of each mouse, and the staining was quantified as fraction of immune-positive staining to the total area measured.

#### Fluorescence Microscopy

The fixed brain sections were blocked with 3% BSA for 30 min, exposed to the anti-cleaved caspase-3 antibody (1:100, Abcam, ab13847) overnight at 4°C and then placed in a wet box containing a little water. After incubation, the sections were washed twice with ice-cold PBS and incubated with Alexa Fluor 594-conjugated goat anti-rabbit IgG (1:250) for 50 min at 37°C in dark. They were then incubated with DAPI solution (Sangon Biotechnology, Co. Ltd., Shanghai, China) at room temperature for 10 min. Immunofluorescence images were acquired using a confocal laser microscope (Leica, TCS SP5, Germany).

#### Thioflavin-T Staining

Thioflavin-T is a kind of fluorochrome that specifically binds to amyloid deposits, and they can be excited to produce green fluorescence, which is used to evaluate the amount of Aβ protein (Zhang et al., 2013). Briefly, slides were deparaffinized and rehydrated in descending grades of ethanol, placed in Mayer’s hematoxylin for 5 min, rinsed twice in double distilled water, incubated with 1% thioflavin-T (Dalian Meilun Biological Technology Co. Ltd., China) for 10 min and changed distilled water three times, cover-slipped in a neutral glycerol, and examined with a Hg-lamp for fluorescence excitation using a Zeiss inverted microscope (Axiovert 200, Carl Zeiss, USA).

### Brain Aβ Enzyme-Linked Immunosorbent Assay (ELISA)

Brain tissues were homogenized in PBS containing 1% SDS and a protease inhibitor cocktail (Roche), and were centrifuged at 10,000 g for 1 h at 4°C. Then, the supernatant was collected as SDS-soluble fraction. In the meantime, the pellets were resuspended and homogenized in 70% formic acid. After centrifugation, the supernatant was collected as the formic-soluble (insoluble) fraction and neutralized with 1 M Tris buffer (pH = 11). Aβ1–40 and Aβ1–42 levels in the brain were determined using Aβ1–40 and Aβ1–42 ELISA kits purchased from the Jiancheng Bioengineering Institute (Nanjing, China) according to the manufacturer’s instructions. The protein concentrations of supernatant were tested using a commercial bicinchoninic acid (BCA) kit (Beyotime Biotechnology, Hangzhou, China) and the normalized amounts of Aβ were expressed as pg/mg of protein.

### Malonaldehyde (MDA) Contents in the Plasma and Brain

The MDA content level in the plasma and brain were measured using a commercial kit (Jiancheng Bioengineering Institute, Nanjing, China) according to the manufacturer’s instructions. Brain tissues were homogenized in PBS and centrifuged at 3,000 rpm for 20 min at 4°C. The supernatant was collected and stored at −80°C until use.

### The Cyclic Adenosine Monophosphate (cAMP) Assay

Cyclic adenosine monophosphate (cAMP) levels in the hippocampus were measured using commercial ELISA kits (Elabscience Biotechnology Co., Ltd, China). Mice hippocampus tissues were homogenized in PBS, and then the homogenates were centrifuged at 5,000 g for 15 min at 4°C to remove the particles. The protein concentrations of supernatant were tested using BCA Protein Assay Kit and the cAMP levels were expressed as pmol/mg protein.

#### Western Blot

Western blot analysis was conducted as described previously (Guo et al., 2015). Protein extraction kit was purchased from KeyGEN BioTECH, Co., Ltd. (Jiangsu, China). The protease inhibitor, phosphatase inhibitor and PMSF were added into RIPA buffer before homogenizing the brain tissues. Protein concentrations were measured using a BCA Protein Assay Kit, and equal amounts of protein (40 μg) were separated by sodium dodecyl sulfate-polyacrylamide gel electrophoresis (SDS-PAGE) and then transferred onto polyvinylidene difluoride (PVDF) membranes (Millipore, Billerica, MA). PVDF membranes were blocked with 5% skim milk at room temperature for 1 h to avoid nonspecific binding and immunoblots were incubated overnight at 4°C with primary antibodies. The primary antibodies we used in this study are listed in Table 1. After primary antibody binding, horseradish peroxidase-conjugated secondary antibodies were incubated for 1 h at room temperature. Blots were developed by the ECL method, and band intensities were quantified using ImageJ Software.

**Table 1:** Antibodies and conditions used for Western blot analyses.

Antibody	Species	Source	Catalog. No.	Dilution
BACE1	Rabbit polyclonal antibody	Abcam	ab10716	1:5,000
LRP1	Rabbit monoclonal antibody	Abcam	ab92544	1:5,000
NEP	Rabbit monoclonal antibody	Abcam	ab79423	1:5,000
IDE	Rabbit polyclonal antibody	Abcam	ab32216	1:1,000
β-amyloid	Rabbit polyclonal antibody	Abcam	ab62658	1:1,000
p-PKA	Rabbit monoclonal antibody	Abcam	ab75991	1:2,500
PKA	Rabbit polyclonal antibody	Abcam	ab71764	1:500
CREB	Rabbit monoclonal antibody	Abcam	ab32515	1:1,000
p-CREB	Rabbit monoclonal antibody	Abcam	ab32096	1:5,000
BDNF	Rabbit monoclonal antibody	Abcam	ab108319	1:2,000
BAX	Rabbit monoclonal antibody	Abcam	ab32503	1:5,000
Bcl-2	Rabbit monoclonal antibody	Abcam	ab182858	1:2,000
Cleaved caspase-3	Rabbit polyclonal antibody	Abcam	ab13847	1:500
Cleaved caspase-9	Rabbit monoclonal antibody	Abcam	ab184786	1:1,000
β-tubulin	Rabbit monoclonal antibody	Beyotime	AF1216	1:1,000

#### Statistical Analysis

All data are expressed as the mean ± SEM. Student’s *t*-test or analyses of variance (ANOVA) with a *post hoc* Tukey’s test were used to determine statistical differences, and *P* values < 0.05 were considered statistically significant. Statistical analyses were performed using GraphPad Prism 6.0 (GraphPad Software, Inc., La Jolla, CA, USA).

## Results

### Identification of Fatty Acids in RJ Samples

**Figure 1:**
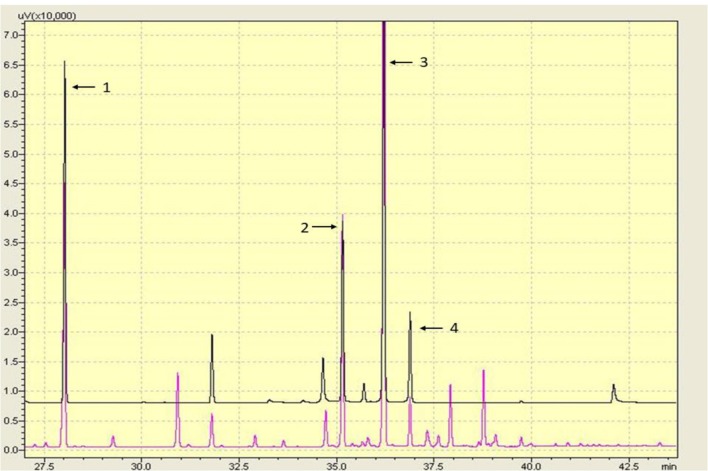
Gas chromatography (GC) chromatography of the lyophilized royal jelly (RJ) powder. Peaks: (1) methyl 4-hydroxybenzoate; (2) 10-hydroxydecanoic acid (10-HDAA); (3) trans-10-hydroxy-2-decenoic acid (10-HDA); and (4) Sebacic acid (SEA).

10-HDA, 10-HDAA and SEA are the main RJ acids, and they were quantified by GC analysis in this study (Figure 1). The percentage of 10-HDA in the RJ was 4.87%, which showed that the RJ used in the present study was of qualified quality.

### RJ Ameliorates Cognitive Deficits in APP/PS1 Mice

**Figure 2:**
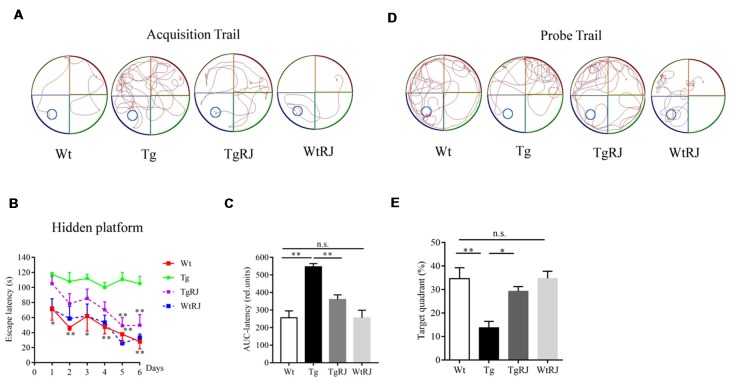
RJ treatment rescued impaired learning and memory function in APP/PS1 mice in the Morris Water Maze (MWM) test. **(A)** Representative path tracking in the acquisition trial with a hidden platform. **(B)** Escape latency in the MWM test plotted against the training days. Data are presented as the mean ± SEM. *P < 0.05, **P < 0.01 compared to the Tg group. **(C)** The area under the curve (AuC) of the escape latency was calculated for statistical comparison. **(D)** Representative path tracking in the probe trial without a hidden platform. **(E)** The percentage of searching time that mice of each group spent in the target quadrant where the platform was located on days 1–6. *n* = 6–10 mice per group. Data are presented as the mean ± SEM. *P < 0.05, **P < 0.01, n.s. non-significant.

In the acquisition trial, mice in the Wt group and WtRJ group could find the submerged platform by a well-organized trajectory after 5 days of training. However, the path tracking of Tg mice was relatively random and disorganized compared with Wt mice. After oral administration of RJ for 3 months, the mice in the TgRJ group had shorter path lengths and selective search tracking, which demonstrated that the memory and learning functions of Tg mice were greatly improved (Figure 2A). The average search times to find the hidden platform (escape latency) of each group during six consecutive days were recorded. As shown in Figure 2B, RJ treatment could shorten the search time of Tg mice and there were significant differences on day 5 and day 6 between the Tg group and TgRJ group (*P* < 0.01). On day 6, the escape latency of the TgRJ group was significantly decreased by 50% compared with the Tg group. In addition, the area under each curve was calculated for quantification of the memory function. The AuC-latency in the Tg group showed a remarkable increase compared to the control values. Compared with the Tg group, a significant decrease in the AuC-latency was observed in the TgRJ group (*P* < 0.01), suggesting that RJ treatment for 3 months could improve memory and learning functions in APP/PS1 mice (Figure 2C). In the probe trial, the percent of time spent in the target quadrant represents the memory retention of the hidden platform location. RJ treatment for 3 months significantly improved memory retention in APP/PS1 mice (Figures 2D,E).

**Figure 3:**
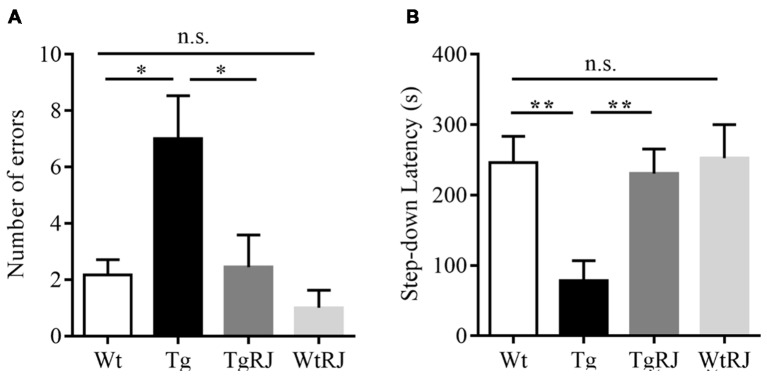
Effect of RJ on the step-down passive avoidance test in APP/PS1 mice. **(A)** The error frequency to step down from a platform after electric shock. **(B)** The latency of the step-down response onto the grid floor 24 h after the training. *n* = 6–10 mice per group. Data are presented as the mean ± SEM. *P < 0.05, **P < 0.01, n.s. non-significant.

The step-down passive avoidance test was carried out the day after the MWM test. The average number of errors in the Tg group was higher than in the Wt group (*P* < 0.05). Moreover, 3 months of oral administration of RJ in APP/PS1 mice significantly reduced the number of errors by more than 3 times (*P* < 0.05; Figure 3A). RJ treatment could reverse the memory function of APP/PS1 mice as reflected by an increased step-down latency (*P* < 0.01; Figure 3B). In addition, there was no difference in the number of errors and step-down latencies between the Wt group and WtRJ group (*P* > 0.05).

### RJ Reduces the Aβ Burden in the Hippocampus and Cortex of APP/PS1 Mice

**Figure 4:**
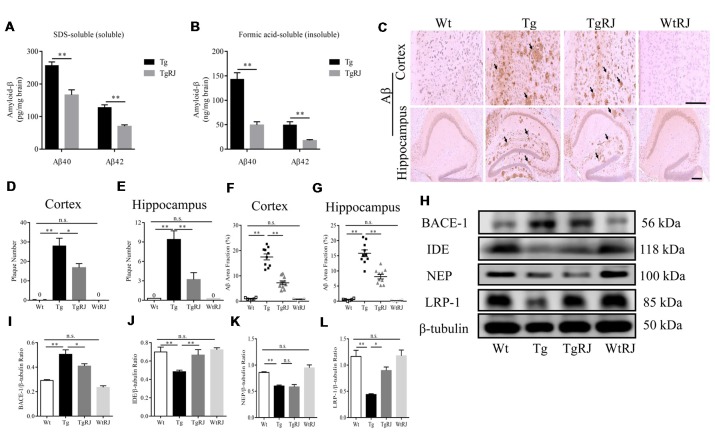
RJ treatment reduced the brain Aβ burden of APP/PS1 mice. **(A,B)** The levels of SDS-soluble and -insoluble (formic acid soluble) Aβ were measured using enzyme-linked immunosorbent assays (ELISAs). **(C)** The Aβ plaques in the cortex and hippocampus were estimated after immunohistochemical (IHC) staining with the Aβ antibody (B-4), the number of plaques per view (**D,E**; cortex, 20× magnification; hippocampus, 40× magnification) and proportions of the positive area **(F,G)** were calculated, three sections per animal. Scale bar = 200 μm. The arrows point out the representative senile plaques (SPs). Data are presented as the mean ± SEM. *P < 0.05, **P < 0.01, n.s. non-significant. **(H–L)** Immunoblot analysis of β-secretase (BACE1), insulin-degrading enzyme (IDE), neprilysin (NEP) and lipoprotein receptor-related protein-1 (LRP-1) in the brain homogenates. *n* = 6–10 mice per group. Data are presented as the mean ± SEM. *P < 0.05, **P < 0.01, n.s. non-significant.

In the SDS-soluble fraction, RJ-treated APP/PS1 mice had significantly less Aβ than the Tg group (*P* < 0.01). The level of Aβ in the brain homogenates of the TgRJ group was reduced by 25% for soluble Aβ40 and 40% for soluble Aβ42 compared with the Tg group levels (Figure 4A). The reduction of RJ on insoluble Aβ levels was more pronounced than the effect on soluble Aβ levels, and the insoluble Aβ40 and Aβ42 levels were reduced by approximately 60% in the TgRJ group (*P* < 0.01; Figure 4B). The amount of insoluble Aβ was much more than the amount of soluble Aβ in the brain of APP/PS1 mice, illustrating that insoluble Aβ is the main form of aggregated Aβ. IHC staining of Aβ also showed that RJ could inhibit the progression of AD by reducing the total area and number of SPs both in the cortex and hippocampus (Figures 4C–G). Western blot and thioflavin-T staining were carried out to further corroborate these findings (Supplementary Figure S2).

To investigate the potential mechanisms responsible for the effect of RJ on Aβ, we examined the expression of BACE1, IDE, NEP and lipoprotein receptor-related protein-1 (LRP-1) using Western blot. The expression of BACE1 was remarkably elevated in the brains of the Tg group compared with the Wt group levels (*P* < 0.01). RJ decreased the expression of BACE1 in the brain homogenates of APP/PS1 mice (*P* < 0.05; Figures 4H,I). Additionally, IHC staining of BACE1 also showed that RJ reduced BACE1 expression by 44% in the cortex (*P* < 0.01) and by 24% in the hippocampus (*P* < 0.05; Supplementary Figures S3A–C). Regarding Aβ-degrading enzyme IDE, RJ treatment upregulated IDE expression at a significant level when compared with the Tg group (Figures 4H,J), and this result was confirmed by IHC staining of IDE (Supplementary Figures S3D–F). However, the expression of another Aβ-degrading enzyme, NEP, was not increased after RJ treatment (Figures 4H,K). The levels of Aβ transport receptors across the blood-brain barrier (BBB), low-density LRP-1 was also measured. LRP-1 expression in the Tg group was significantly decreased compared with the Wt group (*P* < 0.01), and RJ upregulated the protein expression of LRP-1 in the brain homogenates of APP/PS1 mice at a significant level (*P* < 0.05; Figures 4H,L). These results suggested that RJ may reduce the deposition of Aβ in APP/PS1 mice by regulating the production, degradation and clearance process. There was no difference in BACE1, IDE, NEP and LRP1 expression between the Wt group and the WtRJ group (*P* > 0.05).

### RJ Ameliorates Oxidative Stress in APP/PS1 Mice

**Figure 5:**
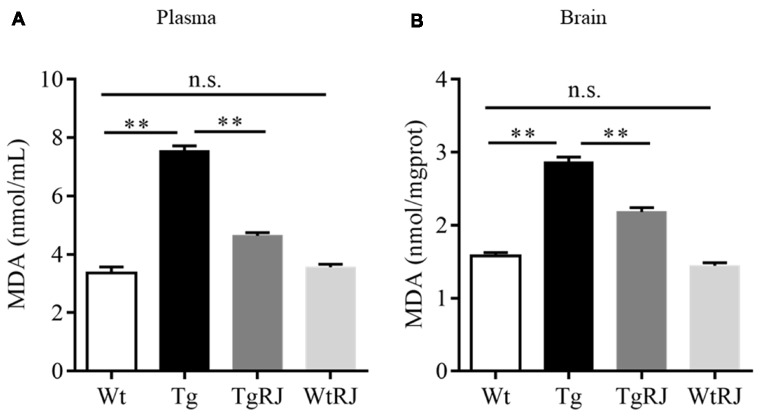
RJ treatment decreased malonaldehyde (MDA) levels in the plasma **(A)** and brains **(B)** of APP/PS1 mice. *n* = 6–10 mice per group. Data are presented as the mean ± SEM. **P < 0.01, n.s. non-significant.

Oxidative stress makes a significant contribution to neurological deterioration, and oxidative damage to lipids, proteins and DNA in the central nervous system occurs in patients suffering from AD (Mancuso et al., 2010). Several lines of investigation have revealed that RJ is a potent anti-oxidative agent (Guo et al., 2009). MDA serves as a sensitive index for evaluating the oxidative stress response and was used as a marker for lipid peroxidation in the brain (Kowalczuk and Stryjeckazimmer, 2002). To determine the effect of RJ on oxidative stress, we tested the MDA levels both in both the plasma and brains of mice. The results showed that the MDA levels in the plasma and brains of the Tg group were significantly increased compared with the Wt group levels, and RJ substantially blunted this increase (Figure 5).

### RJ Inhibits Neuronal Apoptosis in the Hippocampus of APP/PS1 Mice

**Figure 6:**
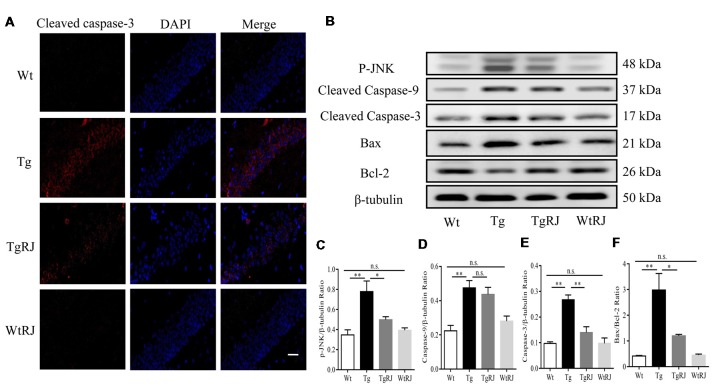
RJ treatment inhibited neuronal apoptosis in APP/PS1 mice. **(A)** Immunostaining of cleaved caspase-3 protein in the hippocampus was performed with a specific primary antibody, and fluorescence was developed using Alexa 594-conjugated anti-rabbit secondary antibody. Scale bar = 40 μm. **(B–F)** The expression of p-Jun N-terminal kinase (JNK), bax, bcl-2, cleaved caspase-9 and cleaved caspase-3 in the hippocampus of mice was detected by Western blot. *n* = 6–10 mice per group. Data are presented as the mean ± SEM. *P < 0.05, **P < 0.01, n.s. non-significant.

Previous studies reported that oxidative stress plays a critical role in neuronal apoptosis (Jiang et al., 2016). Immunofluorescence staining was used to explore whether RJ could alleviate neuronal apoptosis in the hippocampus of APP/PS1 mice. Our results showed that the protein expression of cleaved caspase-3 in the hippocampus of APP/PS1 mice was significantly increased compared with the Wt group but was decreased by RJ treatment (Figure 6A). Neuronal apoptosis was also determined by Western blots, we assessed the expression levels of pro-apoptotic markers including p-Jun N-terminal kinase (JNK), cleaved caspase-3, cleaved caspase-9, bax and the anti-apoptotic marker bcl-2 in the hippocampus of mice. According to our results, RJ treatment could suppress the activation of caspase-3 by inhibiting the phosphorylation of JNK and downregulating the bax/bcl-2 ratio (Figures 6B–F). However, RJ has no effect on the protein expression of cleaved caspase-9 (*P* > 0.05; Figures 6B,D).

### Effect of RJ on the cAMP/PKA/CREB/BDNF Pathway in the Hippocampus of APP/PS1 Mice

**Figure 7:**
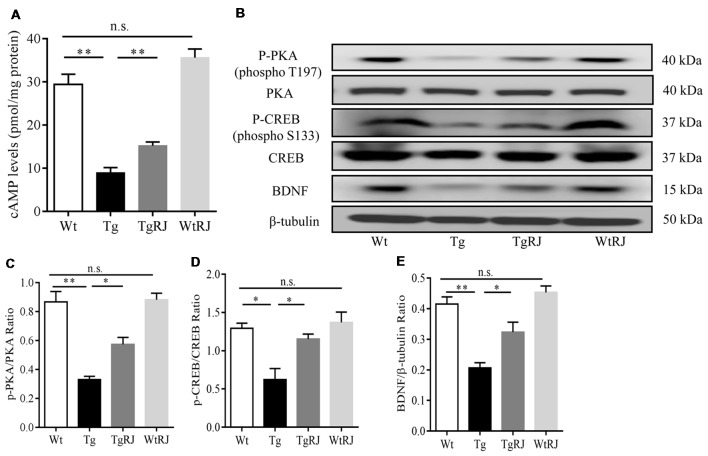
RJ treatment stimulated the cAMP/PKA/CREB/BDNF pathway in the hippocampus of APP/PS1 mice. The protein expression levels of cAMP, p-PKA, PKA, p-CREB, CREB and brain-derived nerve factor (BDNF) were examined using Western blot **(A–E)**. *n* = 6–10 mice per group. Data are presented as the mean ± SEM. *P < 0.05, **P < 0.01, n.s. non-significant.

To investigate the effect of RJ on the cAMP/PKA/CREB/BDNF pathway in the hippocampus of APP/PS1 mice, we examined the changes in cAMP, p-PKA, t-PKA, p-CREB, t-CREB and BDNF protein expressions via ELISA and Western blot assays. Our results showed that, compared with the Wt group, cAMP, p-PKA, p-CREB and BDNF levels were significantly decreased in the Tg group. However, 3 months treatment with RJ remarkedly stimulated this pathway by increasing cAMP, p-PKA, p-CREB and BDNF levels without changing t-PKA and t-CREB levels (Figure 7).

## Discussion

APP/PS1 mice were structured based on Aβ pathology, learning-memory deficits accompanied by detectable cerebral Aβ at 4 months, significant amyloidosis at 6–7 months, and further aggravates pathology at 10 months (Blanchard et al., 2003; Jankowsky et al., 2004). Hence, in the present study, 10-month-old APP/PS1 mice were used as AD models. We conducted MWM test after 2 months of administration RJ and the results showed that spatial learning and memory functions were impaired in APP/PS1 mice, which was in accordance with previous studies (Liu et al., 2014). However, the learning and memory functions of RJ-treated APP/PS1 mice showed no significant improvement compared with saline-treated APP/PS1 mice (Supplementary Figure S4). Thus, we prolonged the RJ-treated time to 3 months. The hippocampus, entorhinal cortex and cingulate cortex are three important areas for spatial memory function and transformation of short-term memory to long-term memory, and damage in these regions is associated with memory loss in AD (Khan et al., 2014; López et al., 2014; Chang et al., 2016). Thus, it is possible that RJ exerts a protective effect on learning and memory functions by inhibiting or repairing hippocampal and cortical lesions in AD brains.

The hippocampus and cortex are vulnerable to damage in the brains of AD patients, and this damage can lead to pathologic changes (Fjell et al., 2014). In view of this, we conducted IHC staining to explore whether RJ could work against the deposition of Aβ in the hippocampus and cortex of AD brains. In line with the behavioral studies, we observed that RJ-treated APP/PS1 mice had significantly less Aβ deposition than vehicle-treated APP/PS1 mice both in the hippocampus and cortex. The imbalance of Aβ production and degradation is an important causative factor in AD progression (Zhu et al., 2013). In this light, we examined the APP proteolytic pathway, the Aβ catalytic pathway and the Aβ transportation pathway in this study. Interestingly, our results showed that the protective effect of RJ could be partly attributed to the increased levels of LRP-1. This result is particularly significant as the efflux transport of Aβ across the BBB is the most crucial prerequisite to Aβ clearance (Erickson and Banks, 2013). In future studies, we will use LRP-1 knockout APP/PS1 mice to completely clarify the role of LRP-1 in the RJ-induced clearance of Aβ. BACE1 and γ-secretase are needed for the amyloidogenic pathological processing of APP to generate Aβ fragments (Willem et al., 2015). Our results showed that the expression of BACE1 was greatly increased in APP/PS1 mice compared with Wt mice. However, it has been reported that there are no differences in the production rate of Aβ in the brains of AD patients and cognitively normal controls (Mawuenyega et al., 2010). Our results led to the hypothesis that γ-secretase may be the rate-limiting enzyme and its expression may remain unchanged in APP/PS1 mice when compared with wild-type mice. The underlying mechanisms of this divergence need further exploration.

Aβ strongly correlates with oxidative stress-related pathology, including cellular dysfunction and death, and subsequent cognitive impairment. Oxidative stress increases the amount of APP and then further aggravates AD pathology (Jiang et al., 2016). This study verified that oxidative stress is a therapeutic target of RJ in AD. AMP *N1*-oxide, a unique compound not found in natural products other than RJ, has important neurothropic activity in brain function. It could induce the generation of neurites and inhibit cell growth through adenosine A_2A_ receptor-mediated protein kinase A (PKA) signaling and potentiate the development of astrocytes (Hattori et al., 2010a). Additionally, 10-HDA could easily cross the BBB and exert unique neuromodulatory activity due to its smaller size. Importantly, 10-HDA initiates neurogenesis by neural stem/progenitor cells (Hattori et al., 2007a). Thus, one hypothesis is that AMP *N1*-oxide and 10-HDA are active components in RJ that alleviate cognitive deficits and Aβ accumulation in the APP/PS1 mouse model. In AD brains, activation of c-JNK has been demonstrated in neurons and dystrophic neurites (Shoji et al., 2000). It has been described that JNK signaling induces activator protein (AP)-1-dependent Bax and caspase activation, which results in neuronal apoptosis (Putcha et al., 2003). Inhibition of the JNK pathway significantly reduced the toxicity attributable to Aβ. Our results showed that RJ inhibited JNK-induced neuronal apoptosis, which may be a mechanism underlying the ameliorative effect of RJ on cognitive deficits.

Previous studies reported that cAMP signal transduction is disrupted in AD brains, and cAMP signal transduction disruption is responsible for memory impairment and neuronal loss (Yamamoto et al., 2000). Decreased cAMP levels inhibit the activation of PKA, followed by reduced levels of phosphorylated cAMP response element binding protein (CREB). BDNF, a downstream gene mediated by CREB, is a neurotrophin with well-established properties of promoting neuronal survival (Chen et al., 2017). Aβ at a sublethal concentration downregulates the BDNF signaling in cultured cortical neurons (Tong et al., 2004). Additionally, preclinical reports have described that AD transgenic mouse models show decreased cortical BDNF expression (Peng et al., 2009). Changes in BDNF levels may lead to cognitive deficits (Nieto et al., 2013). Thus, it is possible that RJ alleviated cognitive dysfunction and neuronal apoptosis by elevating BDNF levels. cAMP plays an important part in the immune process, and the imbalance of cAMP in inflammatory cells leads to inflammatory disorders (Aronoff et al., 2004). Certain inflammatory mediators are potent drivers of AD (Wyss-Coray, 2006). Therefore, increased cAMP levels not only promote BDNF expression but also alleviate neuroinflammation, which may underlie RJ’s protective effect on cognitive function. G protein-coupled receptors (GPCRs) are a superfamily of cell surface receptors. The rhodopsin family of GPCRs represent approximately 85% of the GPCR superfamily. The adenosine receptor consists of four subtypes (A_1_, A_2A_, A_2B_, and A_3_) whose role is primarily to regulate cAMP accumulation in a wide variety of tissues (Prosser et al., 2017). AMP *N1*-oxide, a unique compound of RJ, elicited neuronal differentiation of PC12 cells through adenosine A_2A_ receptor-mediated PKA signaling (Hattori et al., 2007b). Thus, we made a preliminary speculation that the unregulation of cAMP pathway may be supported by A_2A_ signal-mediated activation of CREB.

In summary, our data demonstrate that RJ substantially improved cognitive deficits and reduced SPs of APP/PS1 mice via stimulation of the cAMP/PKA/CREB/BDNF pathway and inhibition of neuronal apoptosis. Our findings show that RJ may be a promising medicine for AD and lay the foundation for therapeutic development of AD focusing on natural products.

## Author Contributions

MY and YP conceived and designed the experiments. MY, YC and YP performed the experiments. MY and YL analyzed the data. YP, MY, JS and YW contributed to the reagents, materials and analysis tools. MY, YL, KW and FH wrote the article.

## Conflict of Interest Statement

The authors declare that the research was conducted in the absence of any commercial or financial relationships that could be construed as a potential conflict of interest.

## Abbreviations

AD: Alzheimer’s disease
Aβ: Amyloid-β
SPs: Senile plaques
NFTs: Neurofibrillary tangles
APP: Amyloid precursor protein
BACE1: β-secretase
sAPPβ: Soluble APP peptide-β
CTFβ: C-terminal fragment-β
IDE: Insulin-degrading enzyme
NEP: Neprilysin
RJ: Royal jelly
10-HDA: *Trans*-10-hydroxy-2-decenoic acid
MWM: Morris Water Maze
MDA: Malondialdehyde
LRP-1: Lipoprotein receptor-related protein 1
AP: Activator protein
cAMP: Cyclic adenosine monophosphate
PKA: Protein kinase A
CREB: cAMP-response element binding protein
BDNF: Brain-derived nerve factor
TMT: Trimethyltin
STZ: Streptozotocin
GPCRs: G protein-coupled receptors.
