# Supplementary material for: Royal Jelly Alleviates Cognitive Deficits and β-Amyloid Accumulation in APP/PS1 Mouse Model Via Activation of the cAMP/PKA/CREB/BDNF Pathway and Inhibition of Neuronal Apoptosis
Source: Front Aging Neurosci. 2019 Jan 4;10:428. doi: 10.3389/fnagi.2018.00428 (PMC6338040; doi:10.3389/fnagi.2018.00428)

**Figure S1**: A flowchart of the experimental schedule used to assess the effects of RJ on AD in APP/PS1 transgenic mice.

**Figure S2**: RJ treatment reduced the brain Aβ burden of APP/PS1 mice according to Western blots and thioflavin-T staining. (A, B) Immunoblot analysis of Aβ in the brain homogenates. (C, D) Brain sections were stained with thioflavin-T, and the number of thioflavin-T-positive plaques per view (40 ×magnification) were calculated, three sections per animal. Scale bar=50 μm. n=6-10 mice per group. Data are presented as the mean ± SEM. **P*<0.05, ***P*<0.01, n.s. non-significant.

**Figure S3**: RJ reduced the brain Aβ burden by down-regulating BACE1 expression and up-regulating IDE expression. (A-C) Representative immunohistochemical staining for BACE1 positive areas in the cortex and hippocampus from mice of each group, and the proportions of the positive areas were calculated. (D-F) Representative immunohistochemical staining for IDE positive areas in the cortex and hippocampus from mice of each group, and the proportions of positive areas were calculated. Three sections per animal, n=6-10 mice per group. Scale bar=100 μm. Data are presented as the mean ± SEM. **P*<0.05, ***P*<0.01, n.s. non-significant.

**Figure S4**: The effect of RJ on the memory function of APP/PS1 mice after 2-month treatment. ***P*<0.01 compared to the Tg group.

Figure S1


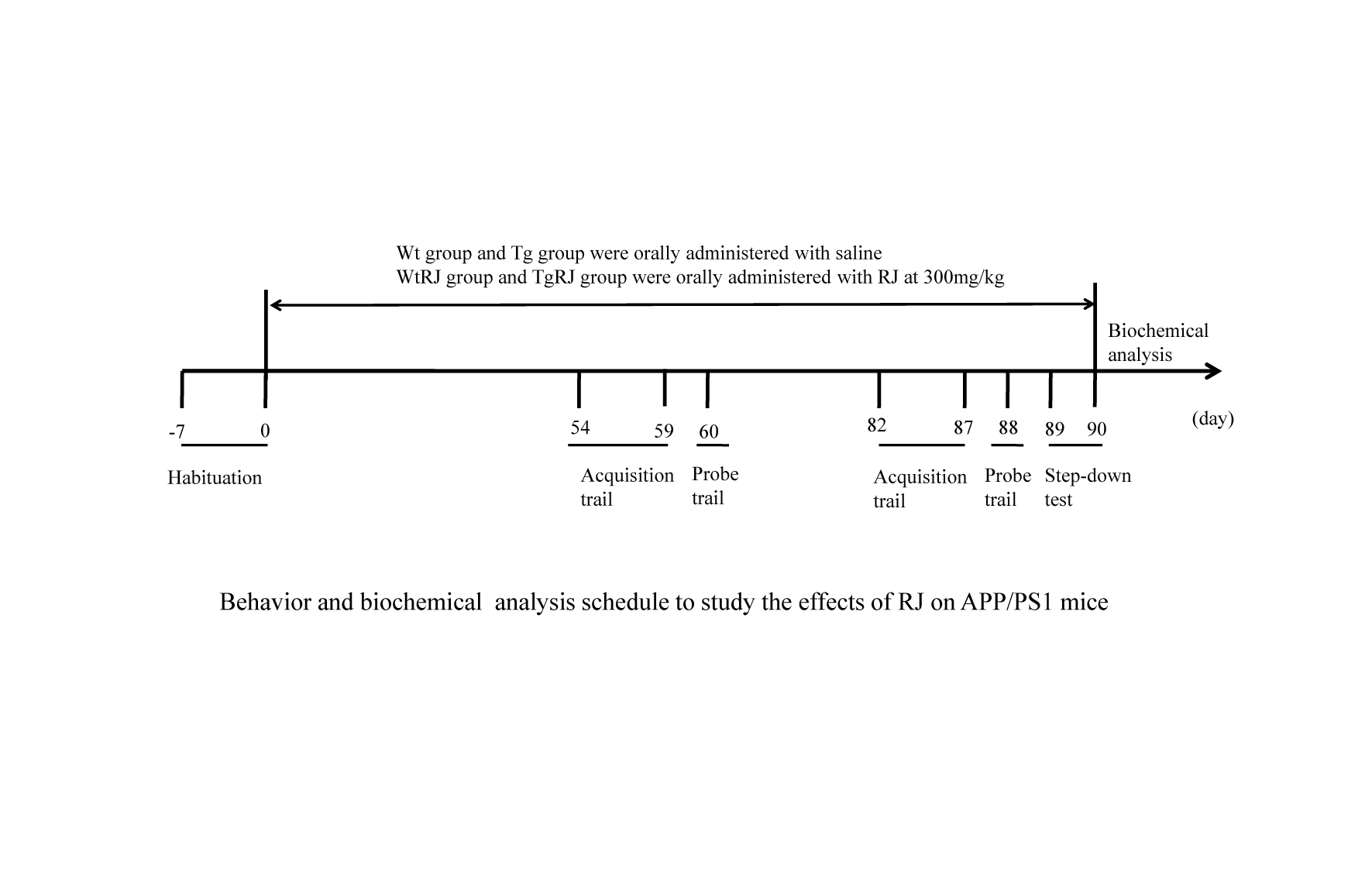


Figure S2


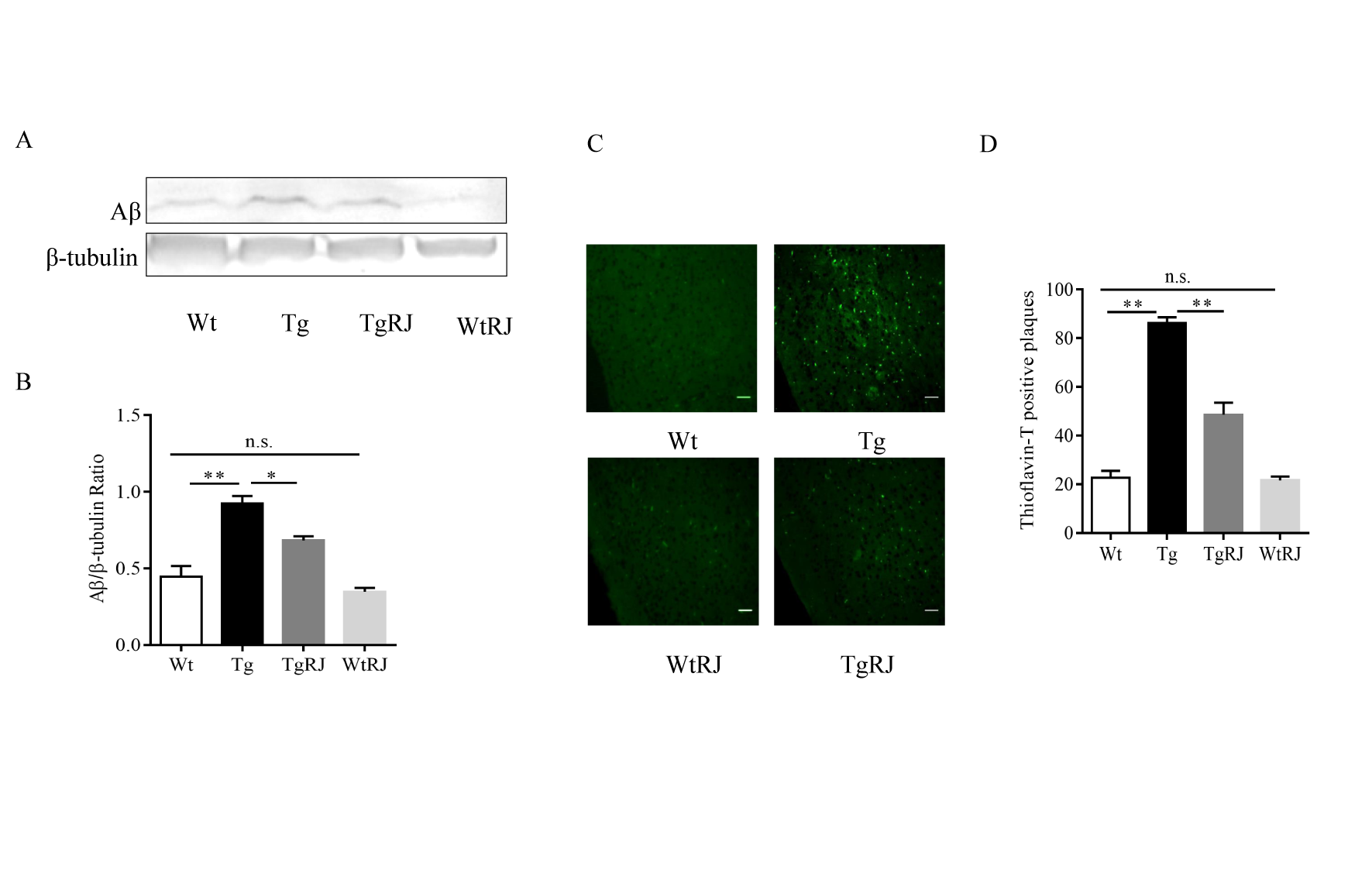


Figure S3


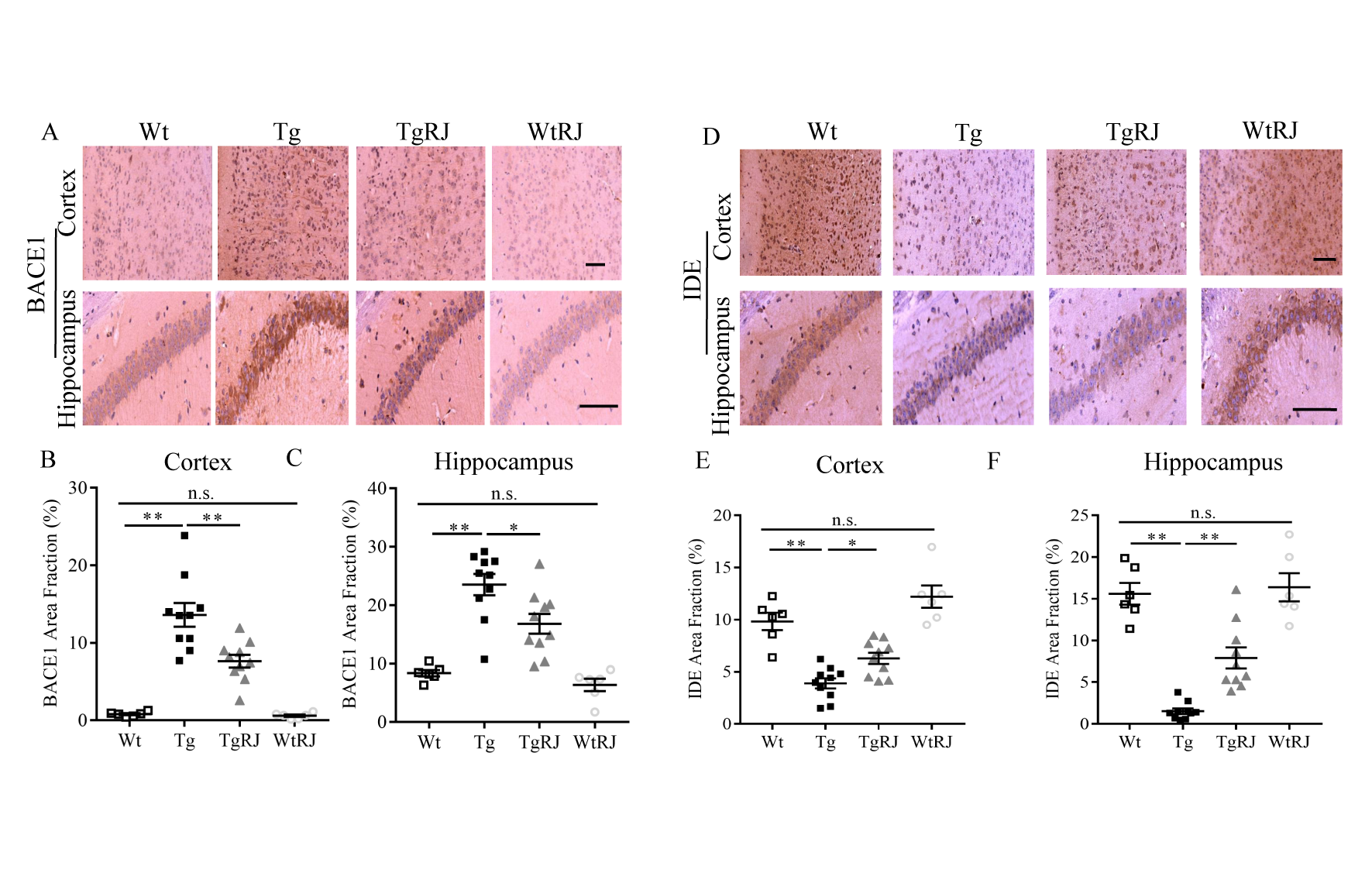


Figure S4


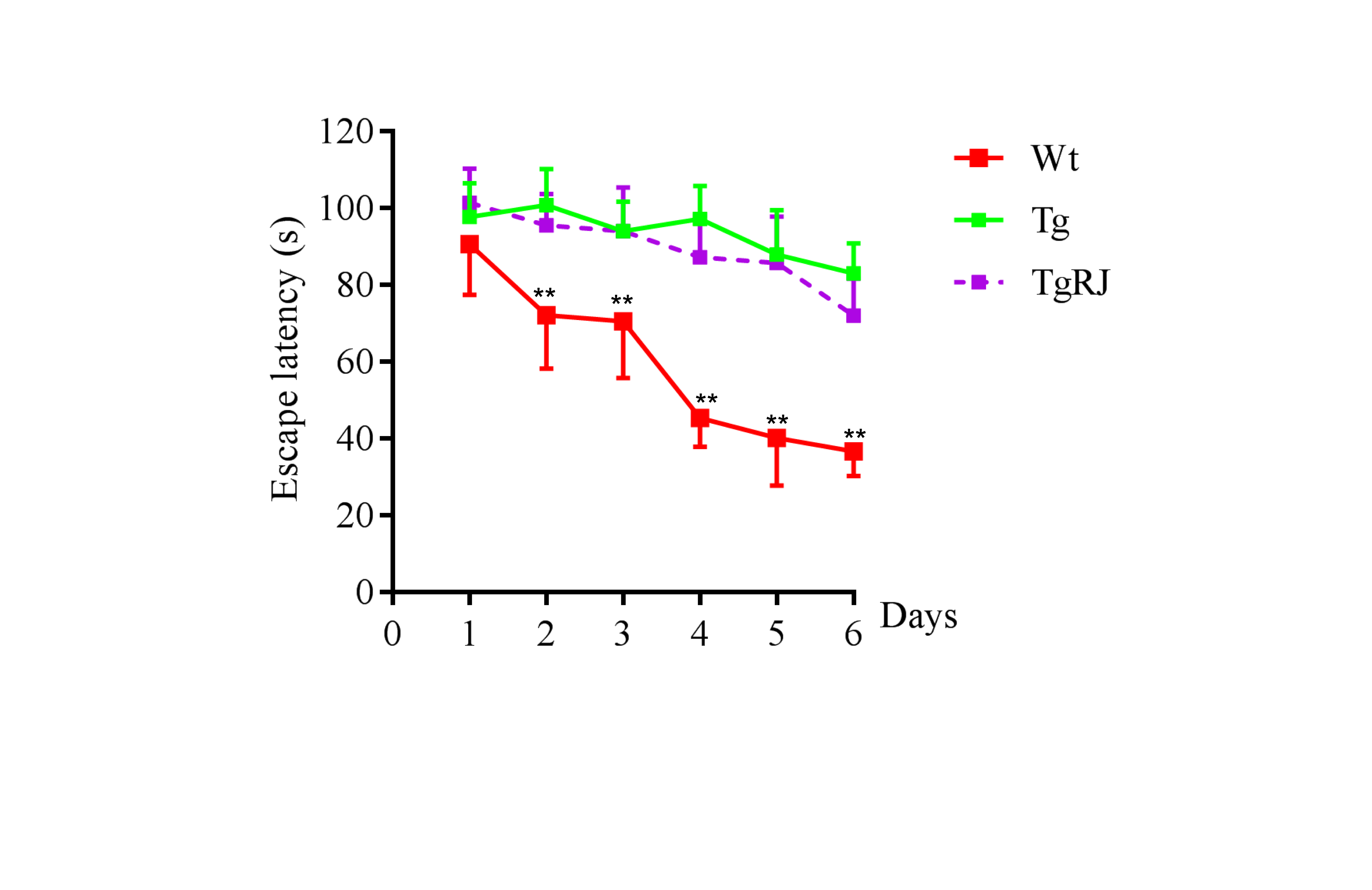

Supplement: Supplementary file 1 [file Data_Sheet_1.doc]
